# Physicochemical Properties and Biocompatibility Evaluation of Collagen from the Skin of Giant Croaker (*Nibea japonica*)

**DOI:** 10.3390/md16070222

**Published:** 2018-06-29

**Authors:** Yunping Tang, Shujie Jin, Xiaoyan Li, Xiaojuan Li, Xuyang Hu, Yan Chen, Fangfang Huang, Zuisu Yang, Fangmiao Yu, Guofang Ding

**Affiliations:** 1 Zhejiang Provincial Engineering Technology Research Center of Marine Biomedical Products, School of Food and Pharmacy, Zhejiang Ocean University, Zhoushan 316022, China; tangyunping1985@163.com (Y.T.); m18868006087@163.com (S.J.); lxj19950329@163.com (X.L.); huxuyang828@163.com (X.H.) cyancy@zjou.edu.cn (Y.C.); gracegang@126.com (F.H.); abc1967@126.com (Z.Y.); 2 Hangzhou Meiya Pharmaceutical Co.Ltd, Hangzhou 310011, China; baishanhou@163.com; 3 Zhejiang Marine Fisheries Reaseach Institution, Zhoushan 316021, China

**Keywords:** *Nibea japonica*, collagen, characterization, biocompatibility, cosmetics

## Abstract

Collagen and collagen peptides are widely used as cosmetic ingredients. In the present study, acid-solubilized collagen (ASC) and pepsin-solubilized collagen (PSC) were extracted from giant croaker (*Nibea japonica*) skin. The proline hydroxylation rates of ASC and PSC were 38.1% and 39.3%. The denaturation temperatures (T_d_) were approximately 34.5 °C for both ASC and PSC. The results of sodium dodecyl sulfate-polyacrylamide gel electrophoresis (SDS-PAGE) and fourier transform infrared spetroscopy (FTIR) demonstrated that ASC and PSC were mainly type I collagen. Furthermore, As, Pb and Hg contents in the extracted collagen were lower than the national standards of China. In addition, collagen had good moisture absorption and retention properties when compared to glycerol. The collagen was also not cytotoxic to NIH-3T3 fibroblast cells, indicating that *Nibea japonica* skin collagen can be utilized in cosmetic applications.

## 1. Introduction

Oceans cover approximately 70% of the earth’s surface, and there are large number of organisms living in the oceans. The marine environment provides a rich source of bioactive substance with excellent and unique cosmetic, functional food or pharmaceuticals products [1,2,3]. Currently, collagen, gelatin, and their hydrolysates (collagen peptide) are widely used as food, pharmaceuticals, cosmetics and biomedicine [4,5]. Bovine and porcine skins or bones are the main sources of collagen and gelatin. However, owing to religious reasons, pig collagen is not ubiquitously used and those extracted from cattle might be contaminated with animal prions [6,7]. Considering the feasibility of collagen as the main ingredient of biomedicine and cosmetics, there is the need for exploring safe and acceptable sources of raw materials for producing collagen. A potential solution to reduce raw materials supply from bovine and porcine may be the by-products from seafood processing. 

Recently, collagen derived from seafood by-products has attracted more attention because of the lack of religious restrictions, low immunogenicity, non-cytotoxicity and high extraction yields [8,9]. Seafood by-products, such as the skin and bone, are abundant in collagen. However, the fish processing industries often discard huge amounts of these by-products and cause the problem of environmental pollution. These low-value raw materials can be transformed into value-added products, with reduced the environmental pollution when using them for extracting collagen. Several collagens have been extracted and characterized from seafood by-products such as bigeye sanpper skin [10], Spanish mackerel skin and bone [6], unicorn leatherjacket skin [7], and Nile tilapia skin [11] and so on. Collagen from seafood by-products can be used as ingredients for cosmetics or biomaterials [12,13]. In addition, extracted collagen is also used for producing bioactive peptides with antioxidant, tyrosinase inhibition, matrix metalloproteinase inhibition or anti-inflammatory activities for cosmeceutical applications [14,15,16,17]. 

The giant croaker (*Nibea japonica*) is now cultured as a promising species for marine aquaculture in East Asia [18,19]. The growth rate of this fish is high (it can reach up to 0.4–0.5 kg in one year) and its price is approximately 6.2 dollars/kg on the Chinese market. During the deep processing of this fish (such as for fish-ball preparation), a large number of by-products such as skin, swimming bladder, bones, and scales are generated, which can be used as a potential source for collagen extraction. However, no studies have provided a systematic comparison of acid-solubilized collagen (ASC) and pepsin-solubilized collagen (PSC) from *Nibea japonica* skin. The application of *Nibea japonica* skin collagen in cosmetics and its cytotoxic and allergenic possibility are also insufficient. Therefore, the physicochemical properties of ASC and PSC were compared, and the biocompatibility of PSC was also evaluated in the present study for further use in cosmetic applications.

## 2. Results and Discussion

### 2.1. ASC and PSC Yields from Nibea japonica Skin

**Figure 1:**
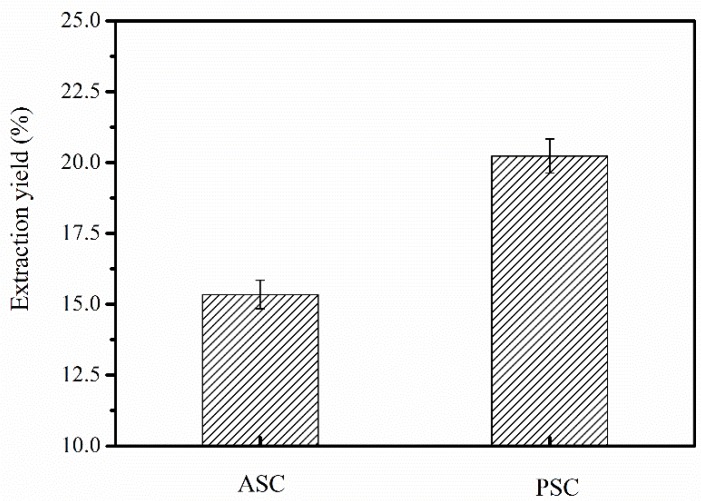
Extraction yield of ASC and PSC from *Nibea japonica* skin (dry weight).

The yields of ASC and PSC extracted from *Nibea japonica* skin were 15.35% and 20.23% (dry weight), respectively (Figure 1). However, the *Nibea japonica* skin was not fully solubilized in 0.5 M acetic acid, which was similar to that observed in previous studies [6,20]. We speculated that the collagen in *Nibea japonica* skin may be cross-linked via covalent bonds among the aldehyde groups at the telopeptide region [21]. The cross-links at the telopeptide region can be cleaved by pepsin, and therefore, the extraction yields of PSC were higher than those of ASC from *Nibea japonica* skin. These results are in agreement with those of Zhang et al. [22], who used pepsin for extracting collagen from frog skin, and Ahmad et al. [7], who used pepsin from tuna stomach for extracting collagen from unicorn leatherjacket skin. Therefore, the above results indicated that pepsin (1200 U/g) combined with acetic acid (0.5 M) can efficiently extract collagen from the skin of *Nibea japonica*.

### 2.2. Amino Acid Composition

The amino acid compositions of *Nibea japonica* skin ASC and PSC are shown in Table 1. The *Nibea japonica* skin ASC and PSC were rich in glycine (Gly), alanine (Ala), proline (Pro), and hydroxyproline (Hyp). However, tryptophan and cysteine were not detected. Furthermore, the Gly content (35.1% and 34.8% for ASC and PSC, respectively,) was similar to that of collagen obtained from other sources [11,23]. This was consistent with the (Gly-X-Y)_n_ repeat structure of collagen polypeptide chains, which is required for assembly into a triple helical structure. In addition, the amino acid content of ASC and PSC was 194 residues/1000 residues and 191 residues/1000 residues, respectively, which was similar to that of Nile tilapia skin collagen (189 and 205 residues/1000 residues) [11], but lower than that of calf skin collagen (215 residues/1000 residues) [22] and human type I collagen (223 residues/1000 residues) [24]. The proline hydroxylation rate was approximately 38.1% for ASC and 39.3% for PSC, which was similar to that of collagen from ocellated puffer fish skin (39%) [25], but lower than that of Nile tilapia skin (41.8% for ASC and 42% for PSC) [11] and channel catfish skin (42.7% for ASC and 42.8% for PSC) [26]. However, since Hyp plays a key role in collagen thermal stability, the difference in proline hydroxylation rate could be species-specific and related to habitat temperature. 

### 2.3. Determination of Denaturation Temperature (Td)

**Figure 2:**
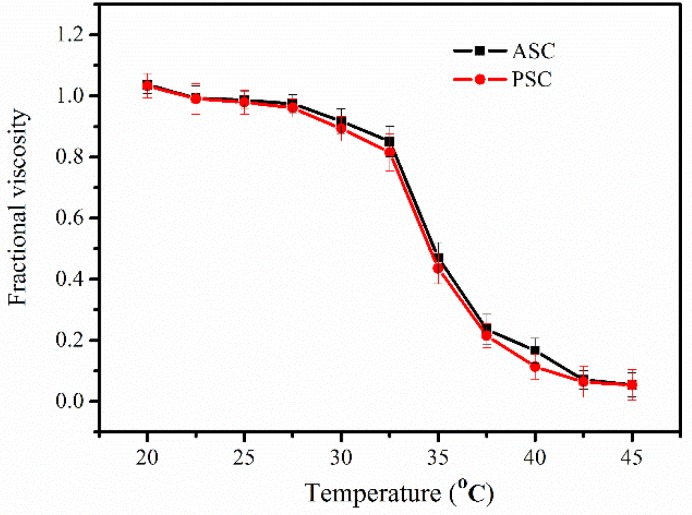
Thermal denaturation curve of ASC and PSC from *Nibea japonica* skin.

The lyophilized ASC and PSC were redissolved in 0.1 M acetic acid, and T_d_ values were determined from the viscosities at different temperatures. The thermal transition curves of ASC and PSC are shown in Figure 2. ASC and PSC showed similar transition curves, and their T_d_ values were approximately 34.5 °C, which is similar to those of collagen from Nile tilapia (approximately 35.2 °C for ASC and 34.5 °C for PSC) [11]. However, the T_d_ values were higher than those of other marine fish collagen, especially those of deep-sea fishes such as Pacific cod (approximately 14.5 °C for ASC and 16.8 °C for PSC) [27]. The thermal stability of collagen was associated with proline and hydroxyproline contents, with higher proline hydroxylation resulting in better thermal stability and high T_d_ value. These results were also consistent with the amino acid composition mentioned above. 

### 2.4. Sodium Dodecyl Sulfate-Polyacrylamide Gel Electrophoresis (SDS-PAGE) Analysis

**Figure 3:**
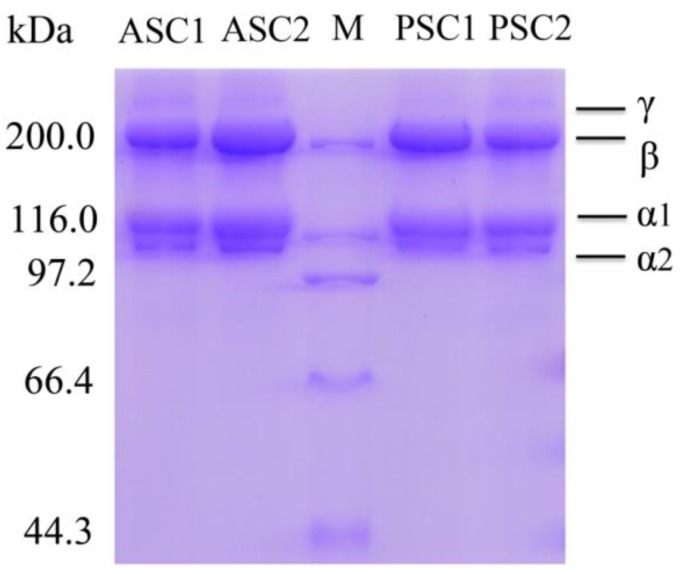
SDS-PAGE analysis of ASC and PSC from *Nibea japonica* skin.M: Protein molecular weight marker; ASC1 and ASC2: ASC extracted from *Nibea japonica* skin; PSC1 and PSC2: PSC extracted from *Nibea japonica* skin.

SDS-PAGE electrophoresis using 7.5% gels was used to determine the protein patterns of ASC and PSC from *Nibea japonica* skin. As shown in Figure 3, ASC and PSC were composed of two distinct α chains (α_1_- and α_2_), some β chains (dimer), and small amounts of γ chains (trimer). The protein patterns of ASC and PSC were similar, showing one band between 116 kDa and 200 kDa, and another band at approximately 116 kDa. As shown in Figure 3, the α_1_-chain density was higher than that of the α_2_-chain, which indicated that the major collagen in *Nibea japonica* skin was of type I ((α_1_)_2_α_2_). Furthermore, the protein pattern of ASC was similar to that of PSC despite excision of the telopeptide region, which did not affect the integrity of the triple helical structure [28]. The γ chain density in ASC was slightly higher than that in PSC, indicating that ASC contained several intramolecular and intermolecular cross-linkages. These results were consistent with those of previous reports on skin collagen of Nile tilapia [11] and Spanish mackerel [6].

### 2.5. Fourier Transform Infrared Spectroscopy (FTIR) Analysis

**Figure 4:**
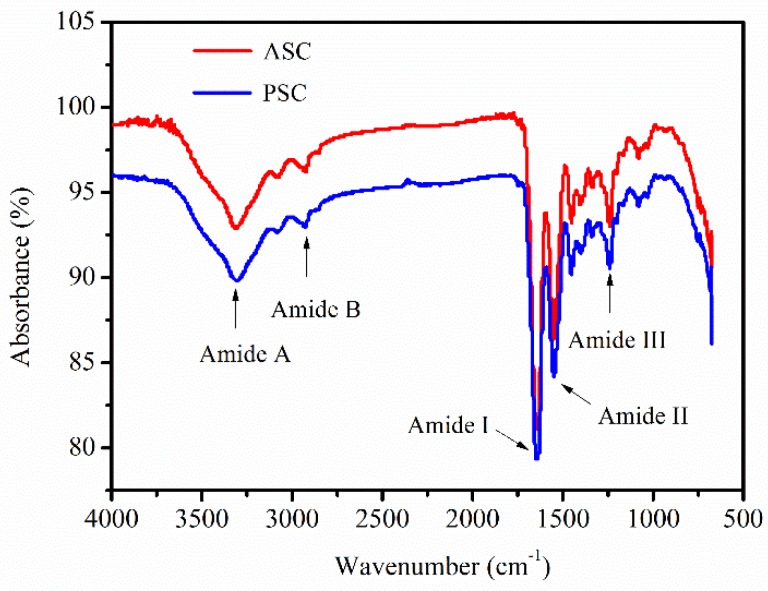
FTIR analysis of ASC and PSC from *Nibea japonica* skin.

The FTIR spectra of *Nibea japonica* skin ASC and PSC is shown in Figure 4. The spectra of ASC and PSC were almost identical, and were similar to those of collagen from other species [29,30]. The peaks contained five main amide bonds, including amide A, B, I, II, and III (Figure 4). The free N-H stretching vibration occurs at wave number ranging from 3400 cm^−1^ to 3440 cm^−1^, with a shift near 3300 cm^−1^ because of the presence of hydrogen bonds [19]. The amide A band, which is associated with N-H stretching frequency, occurred at 3304.82 and 3305.90 cm^−1^ for ASC and PSC, respectively. The amide B bands occurred at 2924.85 cm^−1^ for ASC and 2928.38 cm^−1^ for PSC, which was related to CH_2_ asymmetrical stretching [31]. The amide I bands occurred at 1641.77 cm^−1^ for ASC and 1641.35 cm^−1^ for PSC, which fitted well with the amide I band position (1600–1700 cm^−1^). The amide II bands occurred at 1551.40 cm^−1^ for ASC and 1550.26 cm^−1^ for PSC, which coincided with amide II band position (1550–1600 cm^−1^). Finally, the amide III bands occurred at 1240.29 cm^−1^ for ASC and 1240.47 cm^−1^ for PSC, which indicated the helical structure of collagen extracted from *Nibea japonica* skin [32].

### 2.6. Inductively Coupled Plasma Mass Spectrometry (ICP-MS)

To evaluate the feasibility of using *Nibea japonica* skin collagen in cosmetics or biomedical applications, certain ions were detected using ICP-MS. Element analyses (As, Pb and Hg) of ASC and PSC by ICP-MS are shown in Table 2. The content of As, Pb and Hg in both ASC and PSC was significantly lower than the standard content of China. Collagen from *Nibea japonica* skin does not contain heavy metal ions or other harmful elements, which renders it safe for cosmetics or biomedical applications. 

### 2.7. Moisture Absorption and Retention Properties

**Figure 5:**
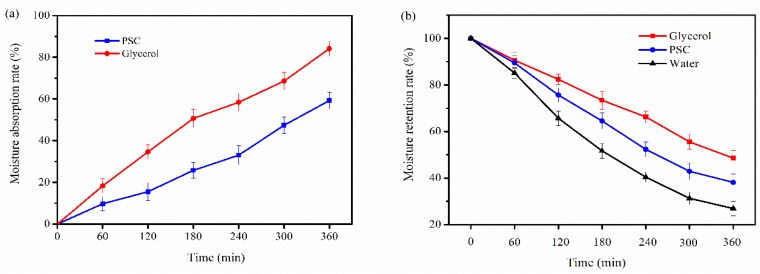
Moisture absorption (**a**) and retention (**b**) properties of PSC.

The moisture-absorption (Ra) and retention abilities (Rh) of PSC from *Nibea japonica* skin were determined in this study. Glycerol, which is commonly regarded as a humectant agent in cosmetics, was used as a positive control. As shown in Figure 5, PSC has good moisture absorption and moisture retention effects when compared to glycerol. The glycerol molecule contains three hydroxyl groups, and its hydrophilic group has a relatively high proportion, so glycerol has the better moisture absorption and moisture retention properties. The PSC extracted from *Nibea japonica* skin has the three helix structure and its exposed hydrophilic group was relatively small, so the moisture retention ability of PSC was lower than that of glycerol. 

### 2.8. Cytotoxic and Allergenic Possibility Test

**Figure 6:**
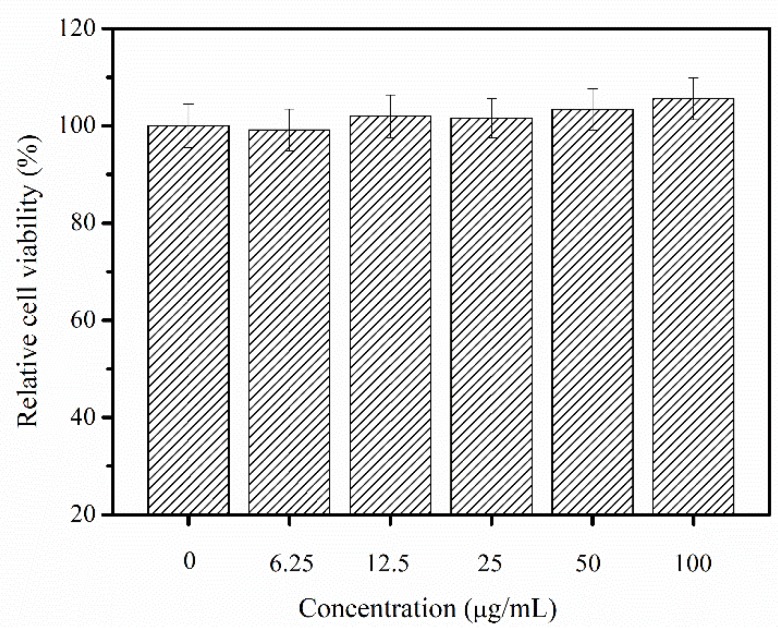
Relative cell viability as affected by 24 h treatment of different concentrations of PSC from *Nibea japonica* skin.

The cytotoxic and allergenic possibility of PSC from *Nibea japonica* skin was evaluated by using MTT assay and LDH toxicity assay. As shown in Figure 6, the viability of NIH-3T3 cells treated with different concentrations of PSC did not decrease after 24 h incubation. On the contrary, the PSC from *Nibea japonica* skin can promote cell growth of NIH-3T3 cells. Thus, *Nibea japonica* skin collagen was found to have no significant cytotoxic effect in vitro. 

**Figure 7:**
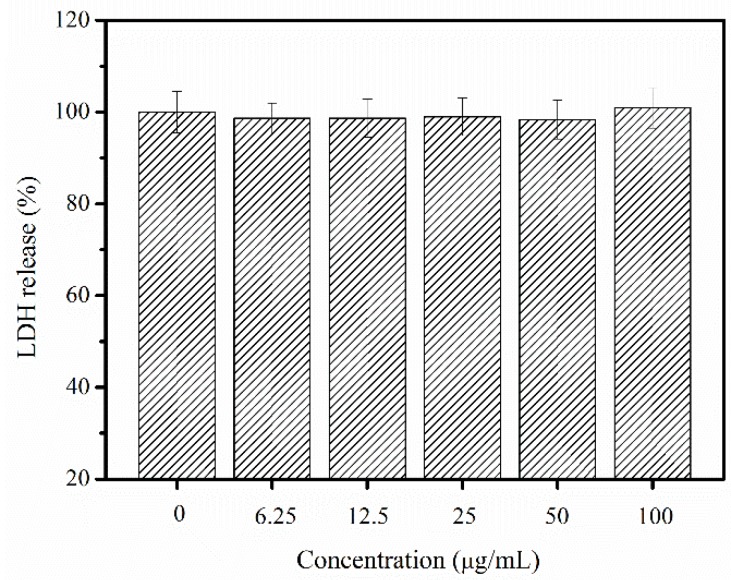
Relative LDH release as affected by 24 h treatment of different concentrations of PSC from *Nibea japonica* skin.

LDH is present in the cytoplasm of all human tissues and the concentrations of LDH will increase as the cellular membrane integrity is disrupted [9]. Therefore, LDH release was reported to be significantly related to the allergic response and inflammation in human study [33,34]. In this study, PSC was found to exhibit lower LDH release than the untreated cells (Figure 7). Therefore, PSC extracted from *Nibea japonica* skin could be potentially regarded as a non-cytotoxic and hypoallergenic material for further use. 

### 2.9. Morphological Examination

**Figure 8:**
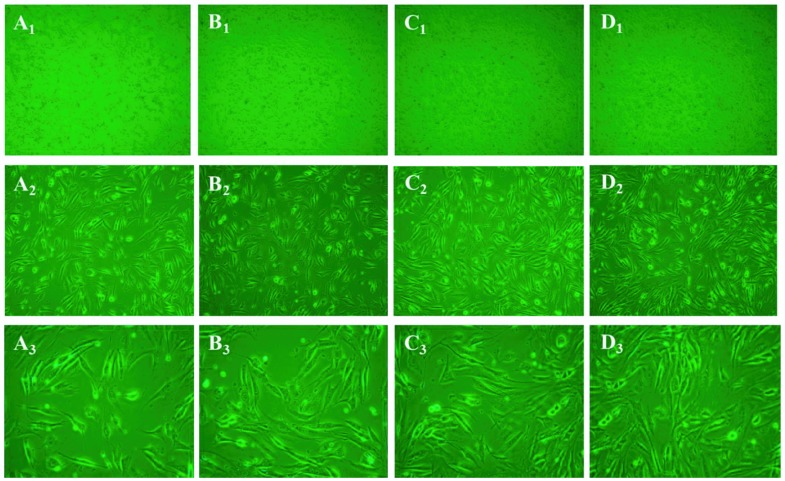
Morphological changes of NIH-3T3 cells treated with different concentrations of PSC from *Nibea japonica* skin (100×, 200×, 400× magnifications). (**A_1_**–**A_3_**) untreated cells under 100×, 200×, 400× magnifications, respectively; (**B_1_**–**B_3_**) treated cells (with 12.5 µg/mL PSC) under 100×, 200×, 400× magnifications, respectively; (**C_1_**–**C_3_**) treated cells (with 25 µg/mL PSC) under 100×, 200×, 400× magnifications, respectively; (**D_1_**–**D_3_**) treated cells (with 50 µg/mL PSC) under 100×, 200×, 400× magnifications, respectively.

Morphological examination of cells treated with different concentrations of PSC (12.5, 25 and 50 µg/mL) indicated no observable changes among cells when compared to the untreated cells (Figure 8). The cells in the experimental groups and control group grew uniformly with normal morphology. As shown in Figure 8, PSC can even promote the cell growth of NIH-3T3 cells, and this phenomenon was consistent with the MTT results. Overall, according to the lack of heavy metals, good moisture absorption and retention, non-toxic and hypoallergenic properties, collagen from *Nibea japonica* skin could be used as a humectant agent in cosmetics. However, further studies such as acute toxicity tests and skin irritation tests on animals will be carried out to demonstrate cosmetic applications. 

## 3. Materials and Methods

### 3.1. Raw Materials

Descaled *Nibea japonica* skin was obtained from the Zhejiang Marine Fisheries Research Institution (Zhoushan, Zhejiang, China) and was stored at −20 °C until use. Pepsin (1:3000 U) was purchased from YTHX Biotechnology Co. Ltd. (Beijing, China). NIH-3T3 fibroblast cell lines were stored in our laboratory. The methylthiazolyldiphenyl-tetrazolium bromide (MTT) cell proliferation and cytotoxicity assay kit, lactate dehydrogenase (LDH) cytotoxicity assay kit were purchased from Beyotime Biotechnology (Shanghai, China). All other reagents were of analytical grade.

### 3.2. Extraction of ASC and PSC

The following steps for extracting collagen were all performed at 4 °C. The descaled fish skins were first mixed with 10 volumes (*v*/*w*) of 0.1 M NaOH and stirred for 24 h to remove the non-collagenous proteins. Then, the alkali-treated fish skins were neutralized and defatted with 10% butyl alcohol overnight. The defatted skins were cut into small pieces and extracted with 0.5 M acetic acid (1:55, *w*/*v*) for 24 h to obtain ASC or with 0.5 M acetic acid and 1200 U/g pepsin (1:55, *w*/*v*) for 8 h to obtain PSC. The ASC and PSC extracts were then filtered, and the filtrates were collected and dialyzed to neutrality with cold distilled water. The final dialysis samples were freeze-dried. The yields of ASC and PSC were calculated by comparing the freeze-dried collagen weight to dried skin weight. 

### 3.3. Amino Acid Analysis

The freeze-dried ASC and PSC were hydrolyzed in 6 M HCl at 110 °C for 24 h without oxygen and then vaporized. The hydrolysates were dissolved in citric acid buffer (25 mL) and analyzed by a Hitachi L-8800 amino acid analyzer (Hitachi, Tokyo, Japan). The content of hydroxyproline (Hyp) was determined according to the chloramine T method described by Sun et al. [11]. 

### 3.4. Determination of Td Values

The Td values of ASC and PSC from *Nibea japonica* skin were determined according to the method described by Sun et al. with slight modifications [11]. The viscosities of ASC and PSC (0.5 mg/mL that dissolved in 0.1 M acetic acid) were determined at several temperatures from 15 °C to 45 °C at an interval of 3 °C. Fractional viscosity at specific temperature was calculated using the equation described by Sun et al. [11].

### 3.5. SDS-PAGE Analysis

The ASC and PSC samples were analyzed using SDS-PAGE electrophoresis [35,36]. The lyophilized ASC and PSC samples (0.5 mg/mL) were redissolved in 0.1 M acetic acid and then mixed with 5× loading buffer. The mixtures were boiled at 100 °C for 3–5 min and the undissolved debris was removed by centrifugation (12,000 rpm, 5 min). Electrophoresis was performed on 7.5% gels and the molecular weights of ASC and PSC were estimated with high protein molecular weight markers. 

### 3.6. FTIR Analysis

The lyophilized ASC and PSC samples (1 mg) were mixed with 100 mg dried potassium bromide and pressed into pellets under dry conditions. The FTIR spectra of ASC and PSC were then determined using an FTIR Tensor 27 spectrometer (Bruker, Rheinstetten, Germany) [22,37]. The spectra were recorded at wavelengths ranging from 4000 cm^−1^ to 500 cm^−1^ at a resolution of 1 cm^−1^ for a single scan. 

### 3.7. ICP-MS Analysis

The Pb, Hg, and As contents in ASC or PSC samples were determined using ICP-MS (Agilent, CA, USA). The samples (0.5 mg/mL) were dissolved in distilled water (35 °C) and then cooled to room temperature. The parameters of ICP-MS were identical to those used by Zhang et al. [22]. 

### 3.8. Moisture Absorption and Retention Properties

The dried samples were placed in a constant temperature and humidity incubator (BS-2FS, Suzhou, China) at 25 °C with 65% relative humidity. The moisture absorption was evaluated as follows: R_a_ (%) = [(W_n_ − W_0_)/W_0_] × 100%. R_a_: the percentage of weight increase of dry sample. W_0_ and W_n_ are the weights of sample before and after they were put into the incubator.

The retention properties of samples were determined as follows: wet samples (containing 10% water) were put into constant temperature and humidity incubator (BS-2FS, Suzhou, China) at 25 °C with 65% relative humidity. The moisture retention was evaluated as follows: R_h_ (%) = (H_n_/H_0_) × 100%. R_h_: the percentage of residual water of wet sample. H_0_ and H_n_ were the weights of sample before and after they were put into the incubator. 

### 3.9. Cytotoxic and Allergenic Possibility of Nibea japonica Skin Collagen

The cytotoxicity of collagen to the NIH-3T3 fibroblast cells was evaluated using an in vitro MTT assay with some modifications as described by Tang et al. [35]. Cells were seeded in a 96-well flat-bottomed plate (1 × 10^5^ cells per well) and allowed to adhere to the bottom of the wells for 24 h and treated with different concentrations of PSC (0, 6.25, 12.5, 25, 50 and 100 µg/mL, pH 7.0). The cells were incubated for another 24 h at 37 °C in a 5% CO_2_ incubator. Absorbance values were measured at 490 nm using a microplate reader, and cell growth inhibition (%) was calculated using the follow equation: Cell growth inhibition (%) = [1 − (OD treated/OD control)] × 100%.

The allergenic potential of collagen to the NIH-3T3 fibroblast cells was evaluated using LDH release assay according to the method described by N.M.H. Khong et al. [9]. Cells were seeded in a 96-well flat-bottomed plate (1 × 10^5^ cells per well) and allowed to adhere to the bottom of the wells for 24 h and treated with different concentrations of PSC (0, 6.25, 12.5, 25, 50 and 100 µg/mL). The cells were incubated for another 24 h at 37 °C in a 5% CO_2_ incubator. The cytotoxic activity was evaluated using an LDH toxicity assay kit according to the supplier’s specifications. 

### 3.10. Morphological Changes of Cells Treated with PSC from Nibea japonica Skin

NIH-3T3 fibroblast cells were suspended in the medium with a final concentration of 1 × 10^5^ cells/mL, and cultured for 24 h in a 6-well flat-bottomed plate with a coverslip (20 × 24 mm). The cells were then treated with PSC at final concentrations of 0, 12.5, 25, 50 µg/mL (pH 7.0). The changes in cell morphological were evaluated using an inverted microscope (Olympus, Tokyo, Japan) after 24 h incubation. 

### 3.11. Statistical Analysis

All experiments were performed in triplicate, and results are expressed as mean ± standard deviation (*n* = 3). Experimental data were analyzed by analysis of variance (ANOVA) using SPSS 19.0 software (IBM SPSS Statistics, Ehningen, Germany).

## 4. Conclusions

In this study, ASC and PSC samples were extracted from *Nibea japonica* skin, and the yield of PSC was higher than that of ASC. The proline hydroxylation rate was 38.1% for ASC and 39.3% for PSC. The T_d_ values of ASC and PSC were approximately 34.5 °C. SDS-PAGE and FTIR showed that ASC and PSC were mainly triple-helical type I collagen. Furthermore, results of ICP-MS indicated that *Nibea japonica* skin collagen was free of heavy metals. The good moisture absorption and retention, non-toxic and hypoallergenic properties indicated that collagen from *Nibea japonica* skin could be potentially used as cosmetic or biomedical materials in the future. 

## Figures and Tables

**Table 1:** Amino acid compositions of ASC and PSC from *Nibea japonica* skin (results are expressed as residues/1000 residues).

Amino Acid	ASC	PSC [19]
Aspartic acid	47	43
Threonine	22	20
Serine	33	29
Glutamic acid	75	73
Glycine	351	348
Alanine	130	128
Cysteine	0	0
Valine	22	19
Methionine	13	10
Isoleucine	10	9
Leucine	27	25
Tyrosine	4	3
Phenylalanine	8	6
Histidine	11	8
Lysine	30	30
Arginine	54	51
Proline	119	116
Hydroxyproline	74	75
Imino acid	194	191

**Table 2:** Elemental analysis of ASC and PSC from *Nibea japonica* skin by ICP-MS.

Collagen	Element	Cotent (mg/kg) ^a^	National Standard of Edible Gelatin (mg/kg)
ASC	As	0.55 ± 0.03	≤0.8
	Pb	0.31 ± 0.03	≤50
	Hg	0.86 ± 0.05	≤50
PSC	As	0.42 ± 0.03	≤0.8
	Pb	0.34 ± 0.02	≤50
	Hg	1.05 ± 0.06	≤50

^a^ Average ± SD from triplicate experiments.

## References

[1] Corinaldesi C., Barone G., Marcellini F., Dell’Anno A., Danovaro R. (2017). Marine microbial-derived molecules and their potential use in cosmeceutical and cosmetic products. Mar. Drugs.

[2] Xu X., Wu X., Wang Q., Cai N., Zhang H., Jiang Z., Wan M., Oda T. (2012). Immunomodulatory effects of alginate oligosaccharides on murine Macrophage RAW264.7 cells and their structure-activity relationships. J. Agric. Food Chem..

[3] Sable R., Parajuli P., Jois S. (2017). Peptides, Peptidomimetics, and polypeptides from marine sources: A wealth of natural sources for pharmaceutical applications. Mar. Drugs.

[4] Fu Y., Therkildsen M., Aluko R.E., Lametsch R., Fu Y., Therkildsen M., Aluko R.E., Lametsch R. (2018). Exploration of collagen recovered from animal by-products as a precursor of bioactive peptides: Successes and challenges. Crit. Rev. Food Sci. Nutr..

[5] Venkatesan J., Anil S., Kim S.K., Min S.S. (2017). Marine fish proteins and peptides for cosmeceuticals: A review. Mar. Drugs.

[6] Li Z.R., Wang B., Chi C.F., Zhang Q.H., Gong Y.D., Tang J.J., Luo H.Y., Ding G.F. (2013). Isolation and characterization of acid soluble collagens and pepsin soluble collagens from the skin and bone of Spanish mackerel (*Scomberomorous niphonius*). Food Hydrocoll..

[7] Ahmad M., Benjakul S. (2010). Extraction and characterisation of pepsin-solubilised collagen from the skin of unicorn leatherjacket (*Aluterus monocerous*). Food Chem..

[8] Silva T.H., Moreira-Silva J., Marques A.L.P., Domingues A., Bayon Y., Reis R.L. (2014). Marine origin collagens and its potential applications. Mar. Drugs.

[9] Khong N.M.H., Yusoff F.M., Jamilah B., Basri M., Maznah I., Chan K.W., Armania N., Nishikawa J. (2018). Improved collagen extraction from jellyfish (*Acromitus hardenbergi*) with increased physical-induced solubilization processes. Food Chem..

[10] Kittiphattanabawon P., Benjakul S., Visessanguan W., Nagai T., Tanaka M. (2005). Characterisation of acid-soluble collagen from skin and bone of bigeye snapper (*Priacanthus tayenus*). Food Chem..

[11] Sun L.L., Hou H., Li B.F., Zhang Y. (2017). Characterization of acid-and pepsin-soluble collagen extracted from the skin of Nile tilapia (*Oreochromis niloticus*). Int. J. Biol. Macromol..

[12] Xhauflaireuhoda E., Fontaine K., Piérard G.E. (2008). Kinetics of moisturizing and firming effects of cosmetic formulations. Int. J. Cosmet. Sci..

[13] Coelho R.C.G., Marques A.L.P., Oliveira S.M., Diogo G.S., Pirraco R.P., Moreira-Silva J., Xavier J.C., Reis R.L., Silva T.H., Mano J.F. (2017). Extraction and characterization of collagen from Antarctic and Sub-Antarctic squid and its potential application in hybrid scaffolds for tissue engineering. Mater. Sci. Eng. C Mater. Biol. Appl..

[14] Wang B., Wang Y.M., Chi C.F., Luo H.Y., Deng S.G., Ma J.Y. (2013). Isolation and characterization of collagen and antioxidant collagen peptides from scales of croceine croaker (*Pseudosciaena crocea*). Mar. Drugs.

[15] Schurink M., Berkel W.J.H.V., Wichers H.J., Boeriu C.G. (2007). Novel peptides with tyrosinase inhibitory activity. Peptides.

[16] Kim M.M., Ta Q.V., Mendis E., Rajapakse N., Jung W.K., Byun H.G., Jeon Y.J., Kim S.K. (2006). Phlorotannins in Ecklonia cava extract inhibit matrix metalloproteinase activity. Life Sci..

[17] Kim J.A., Kim S.K. (2013). Bioactive peptides from marine sources as potential anti-inflammatory therapeutics. Curr. Protein Pept. Sci..

[18] Han T., Li X.Y., Wang J.T., Hu S.X., Jiang Y.D., Zhong X.D. (2014). Effect of dietary lipid level on growth, feed utilization and body composition of juvenile giant croaker *Nibea japonica*. Aquaculture.

[19] Yu F.M., Zong C.H., Jin S.J., Zheng J.W., Chen N., Huang J., Chen Y., Huang F.F., Yang Z.S., Tang Y.P. (2018). Optimization of extraction conditions and characterization of pepsin-solubilised collagen from skin of giant croaker (*Nibea japonica*). Mar. Drugs.

[20] Somasundaram T., Anguchamy V., Muthuvel A. (2013). Isolation and characterization of acid and pepsin-solubilized collagen from the skin of sailfish (*Istiophorus platypterus*). Food Res. Int..

[21] Jongjareonrak A., Benjakul S., Visessanguan W., Nagai T., Tanaka M. (2005). Isolation and characterisation of acid and pepsin-solubilised collagens from the skin of Brownstripe red snapper (*Lutjanus vitta*). Food Chem..

[22] Zhang J.J., Duan R. (2017). Characterisation of acid-soluble and pepsin-solubilised collagen from frog (*Rana nigromaculata*) skin. Int. J. Biol. Macromol..

[23] Liu D., Liang L., Regenstein J.M., Peng Z. (2012). Extraction and characterisation of pepsin-solubilised collagen from fins, scales, skins, bones and swim bladders of bighead carp (*Hypophthalmichthys nobilis*). Food Chem..

[24] Myllyharju J., Nokelainen M., Vuorela A., Kivirikko K.I. (2000). Expression of recombinant human type I-III collagens in the yeast *Pichia pastoris*. Biochem. Soc. Trans..

[25] Nagai T., Araki Y., Suzuki N. (2002). Collagen of the skin of ocellate puffer fish (*Takifugu rubripes*). Food Chem..

[26] Liu H.Y., Li D., Guo S.D. (2007). Studies on collagen from the skin of channel catfish (*Ictalurus punctaus*). Food Chem..

[27] Sun L., Li B., Song W., Si L., Hou H. (2017). Characterization of Pacific cod (*Gadus macrocephalus*) skin collagen and fabrication of collagen sponge as a good biocompatible biomedical material. Process Biochem..

[28] Ogawa M., Moody M.W., Portier R.J., Bell J., Schexnayder M.A., Losso J.N. (2003). Biochemical properties of black drum and sheepshead seabream skin collagen. J. Agric. Food Chem..

[29] Wang L., An X., Yang F., Xin Z., Zhao L., Hu Q. (2008). Isolation and characterisation of collagens from the skin, scale and bone of deep-sea redfish (*Sebastes mentella*). Food Chem..

[30] Yan M., Li B., Zhao X., Ren G., Zhuang Y., Hou H., Zhang X., Chen L., Fan Y. (2008). Characterization of acid-soluble collagen from the skin of walleye pollock (*Theragra chalcogramma*). Food Chem..

[31] Muyonga J.H., Cole C.G.B., Duodu K.G. (2004). Fourier transform infrared (FTIR) spectroscopic study of acid soluble collagen and gelatin from skins and bones of young and adult Nile perch (*Lates niloticus*). Food Chem..

[32] Liu H.Y., Han J., Guo S.D. (2009). Characteristics of the gelatin extracted from Channel Catfish (*Ictalurus Punctatus*) head bones. LWT Food Sci. Technol..

[33] Isobe T., Kato Y., Okubo Y., Koga M. (2001). Evaluation of patch testing in atopic dermatitis using commercially available environmental antigens. Allergol. Int..

[34] Faruqi S., Wilmot R., Wright C., Morice A.H. (2012). Serum LDH in chronic cough: A potential marker of airway inflammation. Clin. Respir. J..

[35] Tang Y.P., Yu F.M., Zhang G.M., Yang Z.S., Huang F.F., Ding G.F. (2017). A purified serine protease from Nereis virens and its impaction of apoptosis on human lung cancer cells. Molecules.

[36] Chen J.D., Li L., Yi R.Z., Xu N.H., Gao R., Hong B.H. (2016). Extraction and characterization of acid-soluble collagen from scales and skin of tilapia (*Oreochromis niloticus*). LWT Food Sci. Technol..

[37] Wang J., Pei X., Liu H., Zhou D. (2018). Extraction and characterization of acid-soluble and pepsin-soluble collagen from skin of loach (*Misgurnus anguillicaudatus*). Int. J. Biol. Macromol..

